# Connect MM Registry as a national reference for United States multiple myeloma patients

**DOI:** 10.1002/cam4.2656

**Published:** 2019-11-07

**Authors:** Sikander Ailawadhi, Sundar Jagannath, Mohit Narang, Robert M. Rifkin, Howard R. Terebelo, Kathleen Toomey, Brian G. M. Durie, James W. Hardin, Cristina J. Gasparetto, Lynne Wagner, James L. Omel, Vivek Kumar, Lihua Yue, Amani Kitali, Amit Agarwal, Rafat Abonour, on behalf of Connect MM Registry Investigators

**Affiliations:** ^1^ Mayo Clinic Jacksonville FL USA; ^2^ Mount Sinai Hospital New York NY USA; ^3^ US Oncology Research Columbia MD USA; ^4^ Rocky Mountain Cancer Centers US Oncology Denver CO USA; ^5^ Providence Cancer Institute Southfield MI USA; ^6^ Steeplechase Cancer Center Somerville NJ USA; ^7^ Cedars‐Sinai Medical Center Los Angeles CA USA; ^8^ University of South Carolina Columbia SC USA; ^9^ Duke University Medical Center Durham NC USA; ^10^ Wake Forest School of Medicine Winston‐Salem NC USA; ^11^ Myeloma Research Advocate/Advisor Grand Island NE USA; ^12^ Brigham and Women's Hospital Boston MA USA; ^13^ Celgene Corporation Summit NJ USA; ^14^ Indiana University Indianapolis IN USA

**Keywords:** multiple myeloma, registries, survival

## Abstract

**Background:**

The Surveillance, Epidemiology, and End Results (SEER) database and National Cancer Database (NCDB) show improved overall survival (OS) in patients with multiple myeloma (MM) over the last 15 years. This analysis evaluated the validity of the largely community‐based Connect MM Registry as a national reference for MM.

**Methods:**

Baseline disease characteristics and survival in US newly diagnosed MM patients were examined using the Connect MM Registry as well as SEER and NCDB databases. Baseline characteristics predictive of longer survival in Connect MM were also identified.

**Results:**

As of February 2017, 3011 patients were enrolled in the Connect MM Registry; 2912 were treated**.** Median age at time of MM diagnosis and age range were numerically similar from 2010 to 2015 across all 3 registries; SEER had a higher representation of nonwhite racial groups than that in the other 2 registries. OS rates suggest proportionate improvement with year of diagnosis among the 3 registries. A Cox proportional hazards model suggests that younger age (<65 years) is associated with longer survival (vs ≥75; HR, 0.39; 95% confidence interval, 0.34‐0.46) in the Connect MM Registry. However, sex (HR, 0.91; *P* = .15) and race (black vs white; HR, 0.88; *P* = .21) were not associated with longer OS.

**Conclusions:**

Data from the Connect MM Registry appear to be largely representative of national trends, comprehensive, and reliable representations of the national MM population. Baseline characteristics were comparable, and survival similarly improved over time among the 3 registries.

**ClinicalTrials.gov, identifier:**

NCT01081028.

## Graphical abstract

Survival data from the Connect MM Registry, which is representative of the national MM population, reflect national trends of improvement. This establishes the Connect MM Registry as a reference for MM survival in the US.

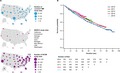

## 1 INTRODUCTION

Outcomes in patients with newly diagnosed multiple myeloma (NDMM) have improved within the past decade. Real‐world data show that the risk of death was 35% lower in patients diagnosed with NDMM in 2011‐2014 compared with those diagnosed in 2006‐2010.^1^ The gap in survival between patients with NDMM and matched controls is decreasing at a rate of 3% per year. Patients treated with novel therapies, including lenalidomide and pomalidomide, within 1 year of diagnosis have better survival than patients treated with regimens without novel agents (*P* = .01).^2^ The contributions of novel agents, autologous stem cell transplantation, triplet regimens, and maintenance therapy for improving overall survival (OS) in patients with NDMM have been extensively described.^3^, ^4^, ^5^, ^6^, ^7^ Real‐world evidence, which is derived from real‐world data sources such as patient registries, can help address the unmet need of describing outcomes in NDMM patients from the general population; of which many (40%) would not be eligible for clinical trials.^8^

Cancer databases help researchers understand outcome trends from national samples vs institutional and trial data, for which outcomes may not be generalizable. Although Surveillance, Epidemiology, and End Results (SEER) database and National Cancer Database (NCDB) are widely used to study cancer outcomes and care, these population‐ and hospital‐based registries collect only limited baseline data (eg, comorbidities, performance status), clinical data (eg, specific treatments received, cause of death [in SEER only]), and lack detailed longitudinal follow‐up (eg, treatment sequencing, response, time to recurrence data, safety, health‐related quality of life) of patients.^9^ The population‐based SEER database of the National Cancer Institute provides cancer incidence and survival data from all available cancer registries in an effort to reduce the cancer burden among the US population. SEER collects data on every case of cancer reported from 19 US geographic areas, which cover about 34% of the US population and are intended to be representative of US population demographics, accounting for diversity.^10^ According to the most recent 6 years of SEER data, the 5‐year OS rate for multiple myeloma (MM) is increasing by >2% every year.^11^ The hospital‐based NCDB, jointly sponsored by the Commission on Cancer (CoC) of the American College of Surgeons and the American Cancer Society, is the largest clinical cancer registry for all cancers in the world. It contains more than 34 million data records (almost 4 times the number in SEER) on treatment and outcomes from cancer patients in hospital registries in CoC‐accredited facilities, which comprise approximately 30% of US hospitals. NCDB data, which represent >70% of newly diagnosed cancer cases nationwide, are used to explore trends in cancer care, to create regional and state benchmarks for participating hospitals, and to serve as the basis for quality improvement.^9^, ^12^ Per NCDB, the median OS for patients with active MM increased from 28.6 months in 2003‐2007 to 40.2 months in 2008‐2011 (*P* < .001).^13^

The Connect MM Registry is a large, US, multicenter, prospective observational cohort study designed to examine real‐world diagnostic patterns, treatment patterns, clinical outcomes, and health‐related quality of life (HRQoL) patient‐reported outcomes in patients with NDMM. More than 3000 patients with NDMM at over 250 community, academic, and government sites were enrolled; 84% of these enrolled patients are from community sites, making it possible to study the heterogeneity observed in routine clinical practice. Baseline and longitudinal data collection are more comprehensive than the SEER and NCDB registries. Participation in the Connect MM Registry is voluntary,^14^, ^15^ whereas participation in SEER and NCDB is not–all cancer cases from the participating regions or hospitals must be reported by government (SEER) or CoC (NCDB) mandate. Connect MM Registry data have been used to establish baseline demographic and disease characteristics in patients with NDMM,^14^ highlight differences between these disease characteristics and those of patients enrolled in clinical trials,^8^ describe treatment patterns,^15^ and develop a clinical tool to predict early mortality and long‐term survival.^16^, ^17^ The objective of this analysis was to evaluate the validity of the largely community‐based Connect MM Registry as a national reference for MM by examining baseline disease characteristics and survival outcomes in patients from the Registry, along with descriptions of data from SEER and NCDB. Any inferences about comparisons across the three databases are purely descriptive. Furthermore, baseline characteristics that were predictors of overall survival in the Connect MM Registry were identified.

## 2 PATIENTS AND METHODS

### 2.1 Connect MM Registry design

Adult patients aged ≥18 years who had symptomatic MM diagnosed within 2 months before enrollment (as defined by International Myeloma Working Group Criteria^18^) were eligible for inclusion in the Registry. All participants were required to provide written informed consent upon enrollment, and the Registry was approved by a central institutional review board (Quorum Review IRB, Seattle, WA, USA) or the institutional review board at the individual study site. The Registry comprises 2 cohorts: patients in cohort 1 (n = 1493) were enrolled from September 2009 to December 2011, and patients in cohort 2 (n = 1518) were enrolled from December 2012 to April 2016. The gap in enrollments between cohorts was not planned, because the decision to begin enrollment for cohort 2 was made 1 year after completion of enrollment for cohort 1. This study is registered at ClinicalTrials.gov as #NCT01081028.

To minimize enrollment bias, enrollment was competitive, and consecutive MM patients presenting to the sites were evaluated for potential enrollment; median time from diagnosis to enrollment was 25 days. All medical treatment (including medications, follow‐up, and posttreatment laboratory testing) was administered at the treating clinician's discretion.

Patients were followed up for treatment and outcomes until early discontinuation or study end (expected 2024). The primary objectives of the Registry are to (a) describe treatment patterns of care of common first‐line treatment regimens and subsequent therapeutic strategies in clinical practice; (b) describe and characterize the occurrence of second primary malignancies. The secondary objectives are to describe (a) treatment patterns and sequencing in relation to clinical outcomes; (b) differences in effectiveness associated with different treatment regimens and regional differences; (c) the HRQoL of patients and its association with treatment regimens/sequence and clinical outcomes. More specific details on the patient population and study design have been previously described.^14^ The current analysis focuses on data from cohorts 1 and 2.

### 2.2 SEER and NCDB patients with MM

MM patient data were derived from 9 population‐based cancer registries from the SEER‐9 (1975‐2015) areas, which include 9.4% of the US population: San Francisco, Connecticut, Detroit, Hawaii, Iowa, New Mexico, Seattle, Utah, and Atlanta. It is standard practice to use SEER‐9 as the default database for calculation of rates due to most complete data and follow‐up. SEER‐9 registries have been present since the inception of the database with other registries added over time (now up to SEER‐21). Follow‐up data were available through the end of 2015, based on November 2013 submission.^19^ Data analyzed from NCDB were from 64,496 patients with MM (MM being the only malignancy or the first of more than 1 malignancy) diagnosed from 2010‐2015.

### 2.3 Baseline demographics and survival analysis

Baseline demographics, disease characteristics, and survival for treated patients in cohorts 1 and 2 of the Connect MM Registry were broadly compared with SEER and NCDB. Baseline demographic data from 2010‐2015 were described for all 3 registries. Survival rates from the 3 registries (2010‐2015 for Connect MM Registry; 2010‐2014 for SEER and NCDB due to data availability) were also examined; no rigorous inferential comparison was intended. No statistical methods were used for comparison across the three databases as this was not the primary intention of the study.

For the Connect MM Registry, SAS version 9.4 was used to analyze adjusted OS probabilities based on the Cox Proportional Hazards (PH) model using data from 2009 to 2015 (2011‐2015 each analyzed separately; 2009‐2010 were combined due to low cohort 1 enrollment from late start [September] in 2009).^20^ The enrollment period‐based OS curves were adjusted to have identical profiles on these covariates at the start of every period. Adjustment covariates (age group, race, and sex) were analyzed by using the Cox PH model to identify baseline characteristics predictive of longer OS. For SEER, due to use of actuarial methods, relative survival rates for myeloma were analyzed using data from 2010 to 2014 (each year analyzed separately). Relative survival compares the survival of patients diagnosed with cancer with the survival of people in the general population who are the same age, race, and sex and have not been diagnosed with cancer.^21^ For NCDB, expected OS probabilities for MM using data from 2010 to 2014 (2010‐2014 each analyzed separately; 2009‐2010 combined) were analyzed.

## 3 RESULTS

### 3.1 Patient characteristics

**Figure 1:**
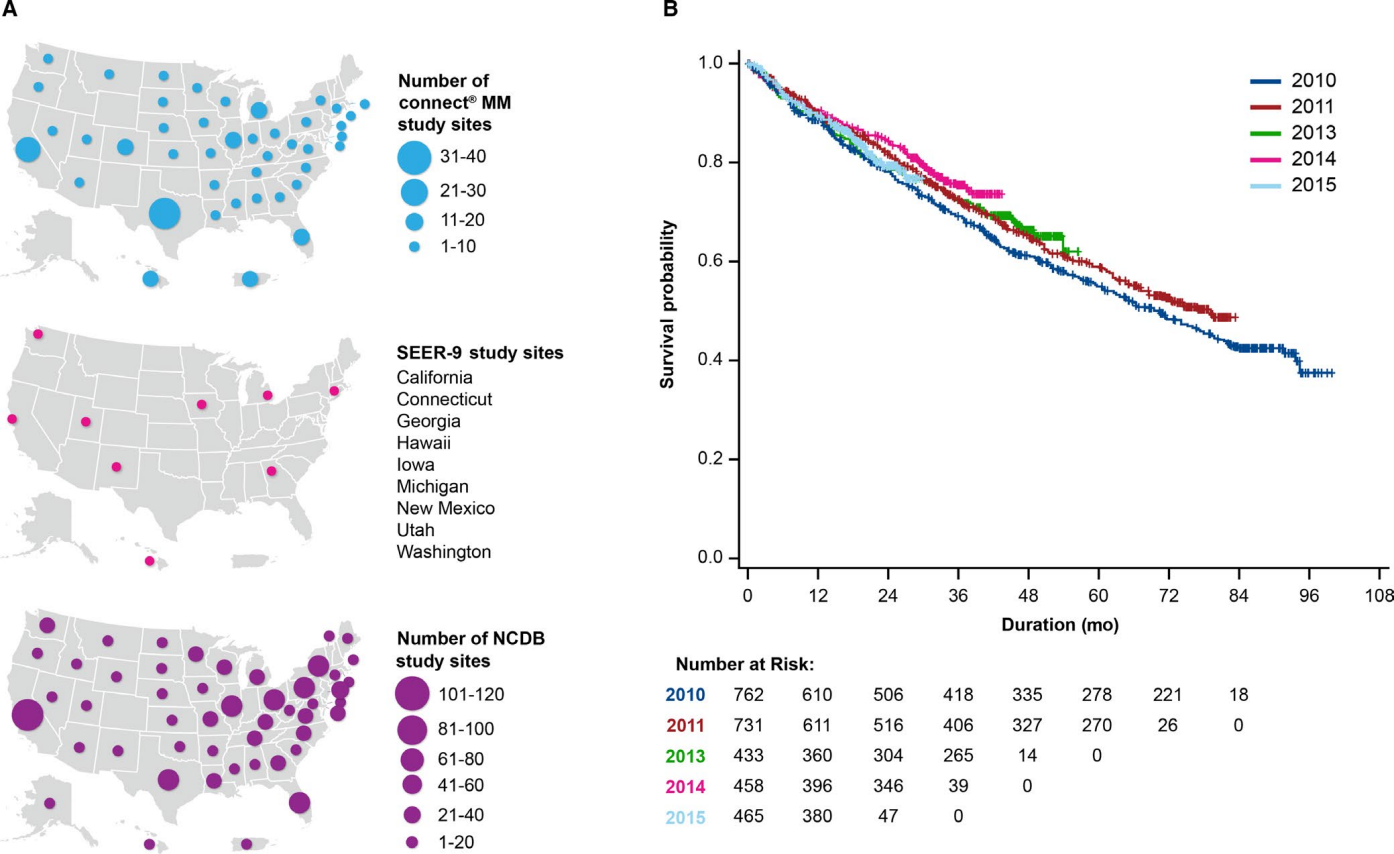
A, Connect MM, SEER‐9, and NCDB sites. B, Connect MM OS by year of enrollment. 2009 and 2010 data were combined. OS was adjusted for the following covariates: age group, sex, and race. MM indicates multiple myeloma; NCDB, National Cancer Database; OS, overall survival; SEER, Surveillance, Epidemiology, and End Results; US, United States.

A total of 3011 patients were enrolled in the Connect MM Registry; 2912 patients were treated. Most patients were from the Midwestern (n = 1080) or Southern (n = 1186) regions of the US; others were from the West (n = 394), Northeast (n = 345), and Puerto Rico (n = 4). Figure 1A depicts maps of sites for all 3 registries. The median age of patients was 67 years (range, 24‐94 years): 1283 patients (42.6%) were aged ≤65 years, 981 patients (32.6%) were aged 65 to 75 years, and 747 patients (24.8%) were aged ≥75 years. The majority of patients were white (n = 2486; 82.6%), 412 (13.7%) patients were black, and 76 patients (2.6%) belonged to other (includes Asian, American Indian/Alaskan Native, Pacific Islander, and Other) racial groups. As of the data cutoff of February 2, 2017, the median duration of follow‐up for all patients who were ongoing or who had discontinued was 65.4 and 24.6 months in cohorts 1 and 2, respectively. A total of 163 patients were lost to follow‐up (n = 108 from cohort 1; n = 55 from cohort 2).

Baseline demographics for the Connect MM Registry, NCDB, and SEER by year of diagnosis are presented in Table 1. Numerically, the Connect MM Registry patient population had a higher proportion of males (53.8%‐61.9% vs 52.8%‐54.8% vs 53.9%‐55.5%) and whites (81.4%‐84.9% vs 67.5%‐69.1% vs 59.4%‐60.5%), and lower proportions of blacks (12.7%‐15.0% vs 20.8%‐21.4% vs 18.8%‐21.5%) than the NCDB and SEER patient populations (respectively) had throughout the enrollment years. Of note, SEER had higher representation of other racial groups (18.4%‐20.8%) compared with the Connect MM Registry (2.2%‐5.9%) and NCDB (9.8%‐11.3%). The median age at the time of diagnosis and age range were similar for all years among all 3 patient populations.

**Table 1:** Baseline demographics of MM patients at time of diagnosis in the Connect MM Registry, SEER, and NCDB

	Enrollment Year	2009/2010	2011	2013	2014	2015
Age, median years (min, max)	Connect MM	67 (24, 93)	67 (27, 94)	66 (33, 91)	67 (32, 94)	67 (34, 94)
	SEER^a^	67 (17, 85+)	67 (17, 85+)	67 (22, 85+)	67 (22, 85+)	67 (17, 85+)
	NCDB	66 (20, 90)	66 (21, 90)	66 (21, 90)	66 (23, 90)	66 (24, 90)
Male, n/N (%)	Connect MM	438/762 (57.5)	416/731 (56.9)	268/433 (61.9)	268/458 (58.5)	250/465 (53.8)
	SEER	4566/8471 (53.9)	2398/4445 (53.9)	2609/4735 (55.1)	2700/4865 (55.5)	2821/5126 (55.0)
	NCDB	9929 (52.8)	5522 (53.0)	6413 (54.2)	6826 (54.8)	6788 (54.3)
White, n/N (%)	Connect MM	620/762 (81.4)	603/731 (82.5)	353/433 (81.5)	389/458 (84.9)	385/465 (82.8)
	SEER	5220/8418 (62.0)	2671/4413 (60.5)	2788/4674 (59.6)	2901/4799 (60.5)	3001/5049 (59.4)
	NCDB	12771/18,470 (69.1)	7117/10,339 (68.8)	8140/11,906 (68.4)	8505/12,433 (68.4)	8408/12,448 (67.5)
Black, n/N (%)	Connect MM	97/762 (12.7)	101/731 (13.8)	65/433 (15.0)	59/458 (12.9)	69/465 (14.8)
	SEER	1647/8418 (19.6)	915/4413 (20.7)	1003/4674 (21.5)	902/4799 (18.8)	1027/5049 (20.3)
	NCDB	3884/18,470 (21.0)	2180/10,339 (21.1)	2546/11,906 (21.4)	2583/12,433 (20.8)	2638/12,448 (21.1)
Other race^b^, n/N (%)	Connect MM	45/762 (5.9)	27/731 (3.7)	15/433 (3.5)	10/458 (2.2)	11/465 (2.4)
	SEER	1551/8418 (18.4)	847/4413 (19.2)	883/4674 (18.9)	996/4799 (20.8)	1021/5049 (20.2)
	NCDB	1815/18,470 (9.8)	1042/10,339 (10.1)	1288/11,906 (10.8)	1345/12,433 (10.8)	1402/12,448 (11.3)

Abbreviations: MM, multiple myeloma; NCDB, National Cancer Database; SEER, Surveillance, Epidemiology, and End Results.

a In the SEER database, median age is provided in 5‐year increments. The calculated midpoints are presented for median age and range.

b For Connect MM Registry, Other race includes Pacific Islander, Asian, American Indian/Alaskan Native, and Other; For SEER, Other includes Hispanic (All Races), non‐Hispanic American Indian/Alaskan Native, and non‐Hispanic Asian/Pacific Islander; For NCDB, Other includes Hispanic, non‐Hispanic Asian, non‐Hispanic Hawaiian/Pacific Islander/others, and non‐Hispanic Native American; Patients with unknown race were excluded.

### 3.2 Survival

In the Connect MM Registry, adjusted OS rates improved with each enrollment year (Figure 1B). Cox PH modeling identified age <65 years (vs ≥75; hazard ratio [HR], 0.39; 95% CI, 0.34‐0.46; *P* < .0001) and age 65 to 75 years (vs ≥75; HR, 0.55; 95% CI, 0.47‐0.64; *P* < .0001) as associated with longer OS. Sex (female vs male; HR, 0.91; 95% CI, 0.80‐1.03; *P* = .15) and race (black vs white; HR, 0.88; 95% CI, 0.73‐1.07; *P* = .21) were not associated with longer OS. Because black and white races accounted for 96% of patients in the analysis, only the HR for blacks vs whites is presented. OS rates suggest proportionate improvement with year of diagnosis among the 3 registries, with longer OS observed in the Connect MM Registry (Table 2).

**Table 2:** Survival rates of MM patients in Connect MM, SEER, and NCDB

Enrollment Year	Database	1‐year Survival (%)	2‐year Survival (%)	3‐year Survival (%)	4‐year Survival (%)	5‐year Survival (%)	6‐year Survival (%)	7‐year Survival (%)
2010	Connect MM^a^	87	76	66	58	51	44	38
	SEER	81.4	72.1	65.5	58.8	53.0	—	—
	NCDB^a^	79.3	68.7	60.3	52.8	—	—	—
2011	Connect MM^a^	89	79	70	61	54	49	49
	SEER	79.8	71.7	64.9	57.7	—	—	—
	NCDB^a^	80.5	70.5	62.6	55.6	—	—	—
2013	Connect MM^a^	88	77	70	62	62	—	—
	SEER	81.3	72.0	—	—	—	—	—
	NCDB^a^	82.5	73.8	65.8	50	—	—	—
2014	Connect MM^a^	89	82	74	74	—	—	—
	SEER	82.8	—	—	—	—	—	—
	NCDB^a^	82.7	73.8	59.7	—	—	—	—
2015	Connect MM^a^	89	77	77	—	—	—	—
	SEER	—	—	—	—	—	—	—
	NCDB^a^	—	—	—	—	—	—	—

Abbreviations: MM, multiple myeloma; NCDB, National Cancer Database; SEER, Surveillance, Epidemiology, and End Results.

a 2009 and 2010 data were combined.

### 3.3 Connect MM Registry cohorts

Baseline characteristics, including age, sex, and race, were similar between cohorts 1 and 2 of the Connect MM Registry (Table S1). Patients with a documented history of monoclonal gammopathy of undetermined significance, smoldering myeloma, or asymptomatic myeloma were uncommon (<11%) in both cohorts.

## 4 DISCUSSION

These results demonstrate that patients from the Connect MM Registry are largely representative of national trends, both in terms of baseline characteristics and overall survival rates. Data from the Connect MM Registry are also more current compared with SEER or NCDB. The median age and age range at the time of diagnosis were similar among all registries. Nonwhite racial groups were better represented in SEER than in the Connect MM Registry and NCDB, owing to the Registry's goal of accounting for diversity in US demographics when selecting geographic areas.^10^ OS rates from 2010 onward similarly improved with year of diagnosis across all 3 large, nationwide registries; though use of relative survival in SEER may result in a small upward bias. Additionally, younger age (<75 years) was predictive of longer OS in the Connect MM Registry. These results add to the body of evidence that patients with MM are indeed living substantially longer than they were over a decade ago.^2^, ^6^ Both Connect MM and SEER collect cause of death (listed as unknown for a fraction of patients), which is of particular importance in this elderly patient population; lack of this information in NCDB is a notable limitation.

Data from the Connect MM Registry are more current and therefore more likely representative of today's real‐world patients with NDMM, compared with data from SEER and NCDB, of which data availability can be delayed for years (eg, the most recent 5‐year relative survival rate for SEER is based on data from SEER 18 that includes years 2008‐2014).^9^, ^21^ Although the sample size of the Connect MM Registry is smaller than that of the MM patient populations in SEER and NCDB, its largely community‐based population (84%) is likely more representative of the broader MM population treated in routine clinical practice.^15^ However, because participation in the Connect MM Registry is voluntary and requires informed consent (vs passive participation in SEER and NCDB), this Registry may have a more knowledgeable and better insured population. NCDB data are derived only from CoC‐accredited hospitals and include data for 70% of newly diagnosed cancer patients. Moreover, sociodemographic variables are skewed toward younger patients (73% of patients <65 years vs 63% of patients ≥65 years) and non‐Hispanics. Thus, results from NCDB analyses may be less representative of smaller community sites. SEER is population‐based; to represent the diversity of the US population, inclusion into the database depends on geographic location. However, not all of the US population is represented in SEER catchment areas, and data for only 30% of newly diagnosed cancer patients are included.^9^ Regardless of these differences, the OS rates are comparable across the 3 registries.

Compared with the Connect MM Registry, data collection in NCDB and SEER is more passive (based on abstraction of medical records) and more limited (eg, lack of comorbidity/specification of comorbidity, time to recurrence, and performance status data).^9^ When describing data from multiple real‐world databases, it is prudent to acknowledge the inevitable risk of patients being concurrently enrolled in more than one of these registries, as they may move from one site to another during the course of their disease and HIPPA requirements for patient deidentification prevent confirmation of patient overlap. The Connect MM Registry focuses on those in the NDMM patient population who were enrolled within 2 months of disease diagnosis, and allows for more complete data on the natural history of MM from diagnosis throughout the patient journey.^14^ NCDB only records the first course of treatment data from patients, of which data collection is delayed for 2 years postdiagnosis in NCDB, and specific treatment data are lacking in SEER with the exception of broad categories (eg, systemic therapy, surgery, and radiotherapy), some of which are not applicable to the definitive management of MM.^9^, ^10^ This Registry collects longitudinal data from patients at each quarter, allowing for various analyses, which have included assessment of second‐line treatment patterns over a 5‐year period,^15^ the effects of posttransplantation maintenance therapy on quality of life, health care resource utilization, and long‐term survival,^22^, ^23^, ^24^ and the incidence of second primary malignancies in patients treated with lenalidomide.^25^

In summary and bearing in mind the previously stated limitations, this analysis demonstrates the utility of the Connect MM Registry—the first, US population‐based registry—as a reference for MM population trends in the US, compared with data from other available national databases.

## CONFLICT OF INTEREST

Sikander Ailawadhi provided consultancy services to Takeda, Novartis, Celgene, and Amgen; he received research funding from Pharmacyclics. Sundar Jagannath provided consultancy services to Celgene, Bristol‐Myers Squibb, Novartis, and Merck; he participated in speakers’ bureaus for MMRF and Medicom. Mohit Narang provided consultancy services and participated in speakers’ bureaus for Celgene; he participated in speakers’ bureaus for Janssen. Robert M. Rifkin provided consultancy services to Amgen, Boehringer Ingelheim, Celgene, EMD Serono, Sandoz, and Takeda; he owns stock with McKesson. Howard R. Terebelo provided consultancy services to Celgene; he participated in speakers’ bureaus for Janssen, Takeda, and Pharmacyclics LLC, an AbbVie Company. Kathleen Toomey provided consultancy services to Celgene, participated in speakers’ bureaus for Myriad Genetics, and received travel reimbursement from Dava Oncology. Brian GM Durie provided consultancy services to Takeda and Janssen. James W. Hardin provided consultancy services to Celgene. Cristina J. Gasparetto received honoraria from Janssen, Bristol‐Myers Squibb, Celgene, and Takeda, provided consultancy services to Janssen, Bristol‐Myers Squibb, and Celgene; she received travel reimbursement from Janssen, Bristol‐Myers Squibb, and Celgene, and received research funding from Celgene. Lynne Wagner provided consultancy services to EveryFit, Gilead, and Janssen. James L. Omel received honoraria from Takeda Oncology and Celgene; he is on the board of directors/advisory committee for Takeda Oncology and Celgene. Vivek Kumar has no conflicts of interest to disclose. Lihua Yue, Amani Kitali, and Amit Agarwal are employed by Celgene. Rafat Abonour is a member of steering committees for Celgene and Takeda, has received research funding from Celgene and Takeda, and has received research funding from Prothena.

## AUTHOR CONTRIBUTIONS

Sikander Ailawadhi: Acquisition, analysis, and interpretation of data, draft review, critical revision for intellectual content, final approval, and accountability for all aspects of the article. Sundar Jagannath: Acquisition, analysis, and interpretation of data, draft review, critical revision for intellectual content, final approval, and accountability for all aspects of the article. Mohit Narang: Acquisition, analysis, and interpretation of data, draft review, critical revision for intellectual content, final approval, and accountability for all aspects of the article. Robert M. Rifkin: Acquisition, analysis, and interpretation of data, draft review, critical revision for intellectual content, final approval, and accountability for all aspects of the article. Howard R. Terebelo: Acquisition, analysis, and interpretation of data, draft review, critical revision for intellectual content, final approval, and accountability for all aspects of the article. Kathleen Toomey: Acquisition, analysis, and interpretation of data, draft review, critical revision for intellectual content, final approval, and accountability for all aspects of the article. Brian G. M. Durie: Acquisition, analysis, and interpretation of data, draft review, critical revision for intellectual content, final approval, and accountability for all aspects of the article. James W. Hardin: Acquisition, analysis, and interpretation of data, draft review, critical revision for intellectual content, final approval, and accountability for all aspects of the article. Cristina J. Gasparetto: Acquisition, analysis, and interpretation of data, draft review, critical revision for intellectual content, final approval, and accountability for all aspects of the article. Lynne Wagner: Acquisition, analysis, and interpretation of data, draft review, critical revision for intellectual content, final approval, and accountability for all aspects of the article. James L. Omel: Acquisition, analysis, and interpretation of data, draft review, critical revision for intellectual content, final approval, and accountability for all aspects of the article. Vivek Kumar: Acquisition, analysis, and interpretation of data, draft review, critical revision for intellectual content, final approval, and accountability for all aspects of the article. Lihua Yue: Acquisition, analysis, and interpretation of data, draft review, critical revision for intellectual content, final approval, and accountability for all aspects of the article. Amani Kitali: Acquisition, analysis, and interpretation of data, draft review, critical revision for intellectual content, final approval, and accountability for all aspects of the article. Amit Agarwal: Acquisition, analysis, and interpretation of data, draft review, critical revision for intellectual content, final approval, and accountability for all aspects of the article. Rafat Abonour: Acquisition, analysis, and interpretation of data, draft review, critical revision for intellectual content, final approval, and accountability for all aspects of the article.

## Data Availability

All CONNECT MM Registry data generated for this analysis are included in this published article. The SEER datasets analyzed during the current analysis are available from the Surveillance Research Program of the National Cancer Institute. These datasets were derived from the following public domain resource: Cancer Statistics Review (CSR), 1975‐2015 [https://seer.cancer.gov/csr/1975_2015/]. The NCDB data that support the findings of this study are available from the following resource: National Cancer Database—Participant User Files [https://www.facs.org/quality-programs/cancer/ncdb/puf].
