# Supplementary material for: Connect MM Registry as a national reference for United States multiple myeloma patients
Source: Cancer Med. 2019 Nov 7;9(1):35–42. doi: 10.1002/cam4.2656 (PMC6943143; doi:10.1002/cam4.2656)
Supplement: Supplementary file 1 [file CAM4-9-35-s001.docx]

**SUPPORTING INFORMATION**

**Table.** Patient Demographics and Clinical Characteristics of the Connect MM Registry

| **Characteristics** | **Cohort 1**  **(n = 1493)** | **Cohort 2**  **(n = 1518)** |
| --- | --- | --- |
|  |  |  |
| **Institutional setting, n (%)** | n = 1450 | n = 1462 |
| Community | 1173 (80.8) | 1281 (87.6) |
| Academic | 259 (17.8) | 166 (11.4) |
| Government | 18 (1.2) | 15 (1.0) |
| **Age** |  |  |
| Median (range), years | 67.0 (24–94) | 67.0 (32–94) |
| < 65 years, n (%) | 649 (43.4) | 634 (41.7) |
| 65 to 75 years, n (%) | 457 (30.6) | 524 (34.5) |
| ≥ 75 years, n (%) | 387 (25.9) | 360 (23.7) |
| **Male sex, n (%)** | 854 (57.2) | 874 (57.6) |
| **Race, n (%)** |  |  |
| White | 1223 (81.9) | 1263 (83.2) |
| Black | 198 (13.3) | 214 (14.1) |
| **History of MGUS, n (%)** | 161 (10.8) | 142 (9.4) |
| **History of smoldering myeloma, n (%)** | 86 (5.8) | 59 (3.9) |
| **History of asymptomatic myeloma, n (%)** | 64 (4.3) | 20 (1.3) |
| **ISS stage (Calculated), n (%)** |  |  |
| I | 334 (22.4) | 303 (20.0) |
| II | 379 (25.4) | 353 (23.3) |
| III | 425 (28.5) | 413 (27.2) |
| **ECOG performance status score, n (%)** |  |  |
| 0 | 351 (23.5) | 320 (21.1) |
| 1 | 526 (35.2) | 456 (30.0) |
| 2 | 140 (9.4) | 117 (7.7) |
| 3 | 34 (2.3) | 44 (2.9) |
| 4 | 2 (0.1) | 1 (0.1) |
| **Lytic bone lesions, n (%)** |  |  |
| 0 | 359 (24.0) | 375 (24.7) |
| 1–3 | 277 (18.6) | 302 (19.9) |
| > 3 | 421 (28.2) | 648 (42.7) |
| **Abnormal serum free light chain level, n/N (%)** | 957/1092 (87.6) | 1085/1198 (90.6) |

Abbreviations: MGUS, monoclonal gammopathy of undetermined significance; ISS, International Staging System; ECOG, Eastern Cooperative Oncology Group.
